# Fit for purpose. Co-production of complex behavioural interventions. A practical guide and exemplar of co-producing a telehealth-delivered exercise intervention for people with stroke

**DOI:** 10.1186/s12961-021-00790-2

**Published:** 2022-01-03

**Authors:** Emily R. Ramage, Meredith Burke, Margaret Galloway, Ian D. Graham, Heidi Janssen, Dianne L. Marsden, Amanda J. Patterson, Michael Pollack, Catherine M. Said, Elizabeth A. Lynch, Coralie English

**Affiliations:** 1 School of Health Sciences and Priority Research Centre for Stroke and Brain Injury, University of Newcastle, Newcastle, Australia; 2 Department of Physiotherapy, Western Health, Furlong Rd, St Albans, Australia; 3 Australian Institute for Musculoskeletal Science, Furlong Rd, St Albans, Australia; 4 Centre for Research Excellence in Stroke Recovery and Rehabilitation, Florey Institute of Neuroscience and Hunter Medical Research Institute, Parkville, Australia; 5 Consumer Partner, Newcastle, Australia; 6 School of Epidemiology and Public Health and School of Nursing, University of Ottawa, Ottawa, ON Canada; 7 Ottawa Hospital Research Institute, Ottawa, ON Canada; 8 Hunter Stroke Services, Hunter New England Local Health District, Newcastle, NSW Australia; 9 School of Health Sciences and Priority Research Centre for Physical Activity and Nutrition, University of Newcastle, Newcastle, Australia; 10 Conjoint Associate Professor School of Health and Medical Sciences, Uni of Newcastle, Newcastle, Australia; 11 Rehabilitation Medicine, John Hunter Hospital, New Lambton Heights, Australia; 12 Centre for Rehab Innovations, Uni of Newcastle, Newcastle, Australia; 13 University of Melbourne, Parkville, Australia; 14 Research Fellow, Adelaide Nursing School, University of Adelaide, Adelaide, Australia; 15 Matthew Flinders Research Fellow, College of Nursing and Health Sciences, Flinders University, Adelaide, Australia

**Keywords:** Stakeholder participation, Integrated knowledge translation, Co-production, Research design, Research partnership, Stroke, Co-design, Intervention development

## Abstract

**Background:**

Careful development of interventions using principles of co-production is now recognized as an important step for clinical trial development, but practical guidance on how to do this in practice is lacking. This paper aims (1) provide practical guidance for researchers to co-produce interventions ready for clinical trial by describing the 4-stage process we followed, the challenges experienced and practical tips for researchers wanting to co-produce an intervention for a clinical trial; (2) describe, as an exemplar, the development of our intervention package.

**Method:**

We used an Integrated Knowledge Translation (IKT) approach to co-produce a telehealth-delivered exercise program for people with stroke. The 4-stage process comprised of (1) a start-up planning phase with the co-production team. (2) Content development with knowledge user informants. (3) Design of an intervention protocol. (4) Protocol refinement.

**Results and reflections:**

The four stages of intervention development involved an 11-member co-production team and 32 knowledge user informants. Challenges faced included balancing conflicting demands of different knowledge user informant groups, achieving shared power and collaborative decision making, and optimising knowledge user input. Components incorporated into the telehealth-delivered exercise program through working with knowledge user informants included: increased training for intervention therapists; increased options to tailor the intervention to participant’s needs and preferences; and re-naming of the program. Key practical tips include ways to minimise the power differential between researchers and consumers, and ensure adequate preparation of the co-production team.

**Conclusion:**

Careful planning and a structured process can facilitate co-production of complex interventions ready for clinical trial.

**﻿Graphical Abstract:**

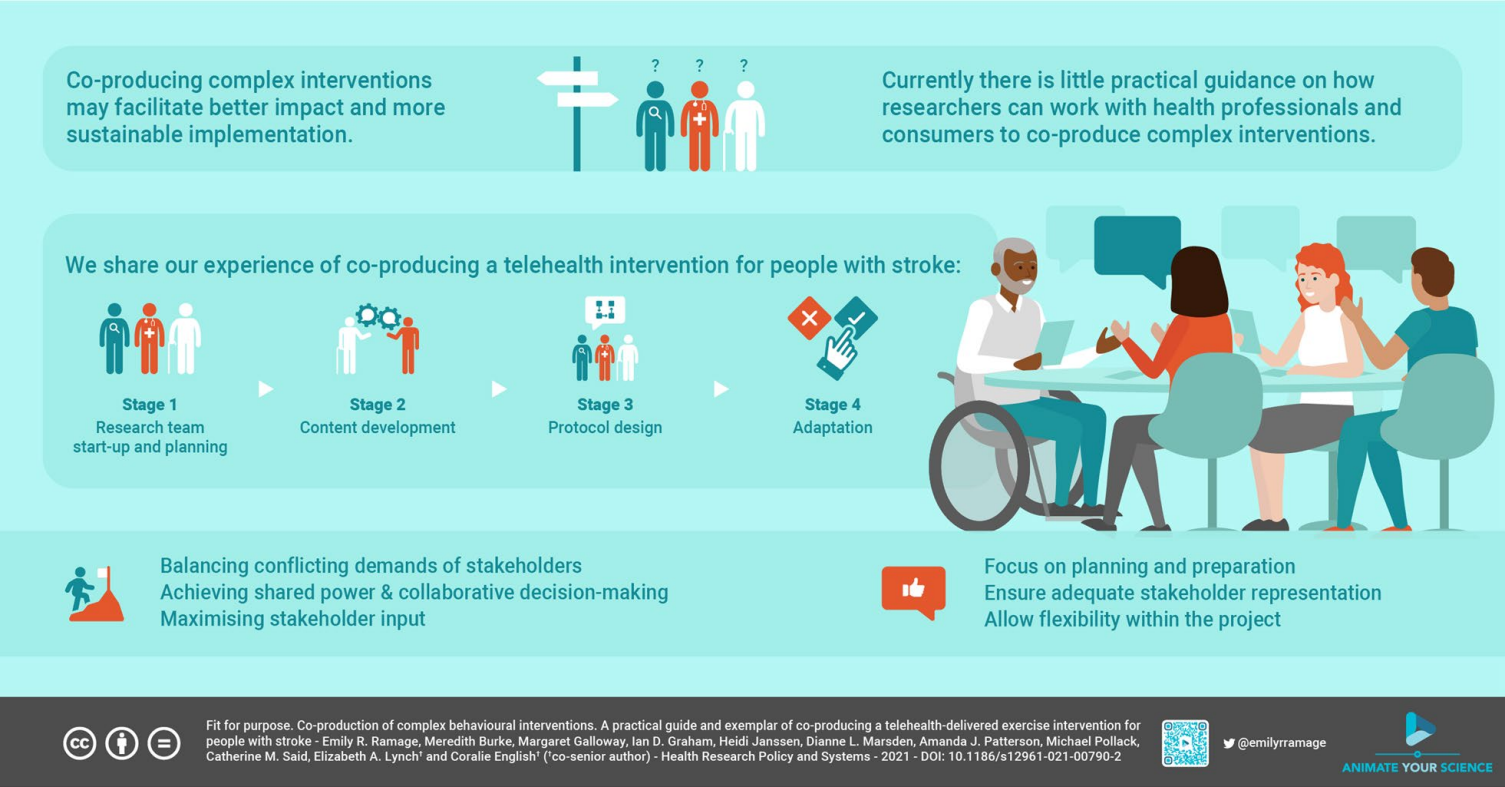

**Supplementary Information:**

The online version contains supplementary material available at 10.1186/s12961-021-00790-2.

## Introduction

Using co-production when developing clinical interventions allows for the perspectives and expertise of different stakeholder groups to be incorporated, thereby enhancing an intervention’s real-world application [1]. Successful healthcare interventions need to be both efficacious and implementable in clinical practice [2]. In the field of stroke recovery and rehabilitation, interventions tend to be complex because they have multiple components and pathway complexity [3]. Poor intervention design and lack of appropriate stakeholder engagement in stroke rehabilitation [4] may be contributing factors to the numerous neutral Phase III clinical trials [5]. Therefore, the Stroke Recovery and Rehabilitation Roundtable expert group have called for improvement in how interventions are developed for clinical trials [5, 6]. Co-production of interventions is important to optimise their success in clinical trial.

There is a lack of consensus of the definition of co-production, for the purpose of this paper we adopt the definition that co-production in research is a “collaborative model of research that includes stakeholders in the research process” [7]. Collaborative research approaches share a number of similarities, one of which is true partnership, i.e. a deep relationship between the researchers and knowledge users throughout the research process to ensure the research benefits all parties [8]. Integrated knowledge translation (IKT) is one co-production research approach in which ‘knowledge users’ are included in the research team in shared partnership [8–10]. Knowledge users are people who will administer, influence, or use the research [9, 11]. Knowledge users can include healthcare workers, policymakers who implement research findings, and people with lived experience of the health condition of interest. IKT has previously been used in research involving policymaking, health promotion, knowledge dissemination, evidence-informed decision making, and the development of implementation or quality improvement interventions [12–16]. However, we could not find any published examples that provide practical guidance regarding the use of an IKT co-production approach to design interventions ready for clinical trial.

The aims of this paper are to:

1. Provide practical guidance for researchers to co-produce interventions ready for clinical trial by describing the 4-stage process we followed, the challenges experienced and practical tips for researchers wanting to co-produce an intervention for a clinical trial;
2. Describe, as an exemplar, the development of our intervention package.

### The project, its context, contributors and supporting evidence base

Stroke is a leading cause of death and disability worldwide, and most strokes are due to modifiable risk factors (e.g., physical activity, diet, smoking, medication adherence) [17]. Over a quarter of all preventable strokes are secondary strokes [18]. While physical activity is recommended in international guidelines to address secondary stroke risk [19–21] the most effective way to deliver physical activity interventions remains unclear. Maintaining adequate physical activity levels after stroke is challenging, and the majority of stroke survivors do not meet minimum activity targets [22, 23]. A physical activity intervention based on what we know physiologically should reduce stroke risk, that is feasible to deliver, and that is also usable and accessible for stroke survivors is needed.

Our paper describes the IKT co-production of a physical activity intervention as part of the development of the *Secondary Prevention of Stroke: Study Protocol for a Telehealth-Delivered Physical Activity and Diet Pilot Randomized Trial (ENAbLE-Pilot)* [24]. This intervention is currently being tested as part of the ENAbLE pilot trial (trial registration ACTRN12620000189921). An IKT approach to co-production was chosen because of its focus on increasing knowledge use and impact [8], its ability to be applied pragmatically and with the understanding that IKT shares many similarities with other collaborative research approaches [8].

## Method

### Project design

From January to August 2019, we co-produced an intervention ready for clinical trial using an IKT approach and following principles outlined in other key resources regarding collaborative research and intervention design [25–27].

**Fig. 1:**
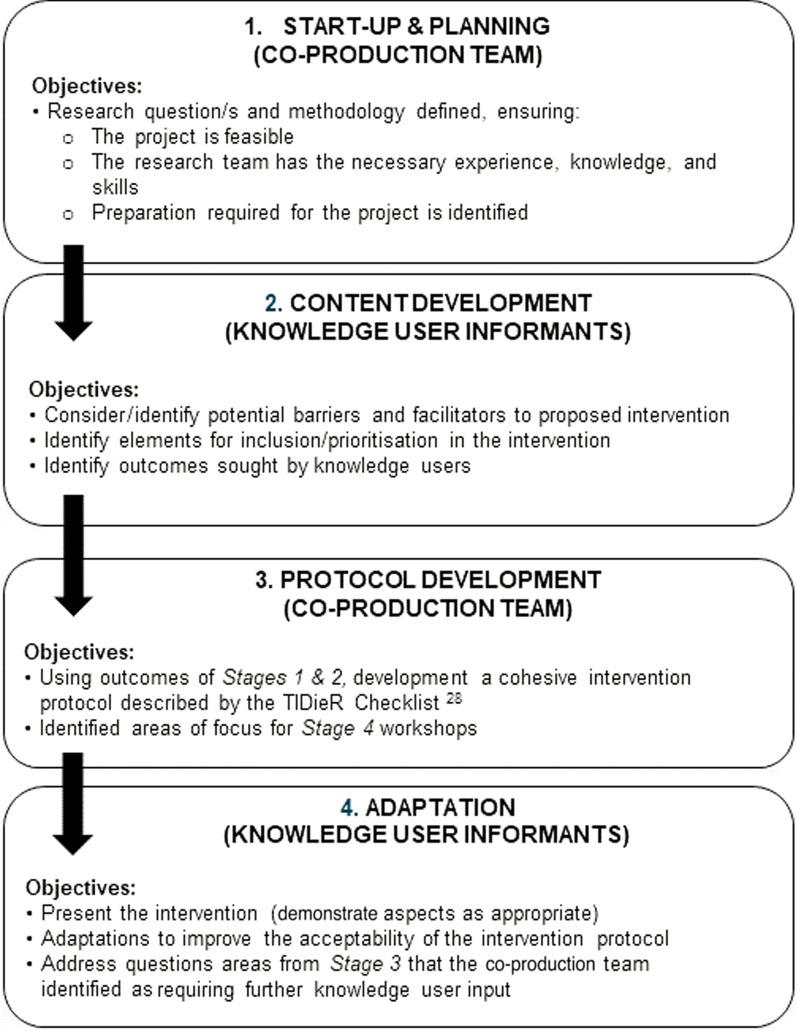
Summary of the four stages of the intervention design process

The co-production was conducted across two states of Australia through a series of workshops and interviews over four stages, summarized in Fig. 1. The Template for Intervention Description and Replication (TIDieR checklist) [28] was used to guide description of the developed intervention.

The investigators in this project were the co-production team. This group included researchers with experience in clinical trials in stroke (CE, CS, EL, ER), and/or cardiovascular risk reduction (CE, AP), implementation science and co-production researchers (EL, IG), clinicians with research training and experience (HJ, DM, MG, MP) and a person with lived experience of stroke (MB). The intervention design process was grounded in an IKT theoretical approach which was used to develop a priori a four-stage framework outlined in Fig. 1. The iterative nature of co-production saw processes evolve over the life of the project, as highlighted in the exemplar presented in the results section of this paper.

We will now describe our four-stage co-production process, including the steps involved in each stage:

#### Stage 1: start-up and planning (January to February 2019)

The start-up and planning phase consisted of 4 main steps. The first step involved assembling the co-production team to lead the project. Our team involved researchers and knowledge users with complementary and relevant skills and experience, including a person with lived experience of the condition for whom the intervention is intended (stroke), and other key knowledge users including clinicians, in our case the clinicians involved also had research experience. The second step was to define the scope of the co-production—such as what essential components of the intervention were essential and non-negotiable and what time frames and resources were available for the project. The third step was to identify the key knowledge user informant groups that needed to be invited to workshops. Knowledge user informants are those who use and apply the intervention (e.g. stroke survivors and healthcare workers), however, they are involved in a consultative role rather than in partnership. Therefore, knowledge user informants provide knowledge and experience to inform the intervention but are not co-producers, as they do not share the power to make key decisions regarding the intervention design as the co-production team does (stages 1 and 3). Finally, the timing and structures of the workshops planned and strategies to optimise the input and engagement of the knowledge user informants was planned. Throughout this stage the co-production team reflected on their plans to help ensure it remained feasible, and feedback on processes and outcomes were welcomed.

#### Stage 2: content development (March to April 2019)

This stage aimed to gather broad perspectives to inform the intervention design. It consisted of separate workshops for different groups of knowledge user informants, i.e., workshops for people with a lived experience of the condition (stroke), and healthcare professionals and other key knowledge user informants including a behaviour change researcher, people working in healthcare management including telehealth management.

The first step was to recruit our knowledge user informants. Recruitment occurred via networks of the co-production team (both our clinician knowledge user and researcher members); social media; websites; contacting previous participants in a telehealth exercise study who consented to being contacted about participating in other research; or, via clinicians at one of the study’s sites. Purposive sampling was used to optimise diversity of knowledge and experience. The second step was to prepare for the workshops; this involved identifying individual needs and preferences of knowledge user informants to optimise their ability to contribute to the workshops. Individual interviews were arranged to accommodate the preferences of some knowledge users who were unable to attend workshops. This step also involved organising logistics (e.g., catering and parking for knowledge user informants).

The third step was to undertake the workshops and summarise their findings. Workshops commenced with an outline of the intent of the overall co-production process, the aims and expectations of the workshop. The facilitator then posed questions to the group to encourage discussion and identification of key elements that should be included in the intervention.

The workshop facilitator (ER) iteratively summarised and confirmed ideas and outcomes throughout workshops and interviews to ensure consensus and accurate interpretation of knowledge user informant input. Workshops were audio- recorded, and notes were taken during the workshops by the facilitator. Following each workshop, a summary was sent to the knowledge user informants involved, who were given the opportunity to make amendments at this time.

#### Stage 3: intervention protocol development (May to June 2019)

The aim of this stage was to incorporate the elements identified in previous stages of the project into a comprehensive protocol prototype. The first step in Stage 3 involved collating the elements knowledge user informants identified as important to be included in the intervention design (data source stage 2 workshops summaries) with the essential components of the intervention determined previously by the co-production team (as determined in stage 1) into a summary document. This document mapped these elements into the Template for Intervention Description and Replication (TIDieR) Checklist. When designing interventions ready for clinical trial the outcomes to be measured must be considered, however this is not captured in the TIDieR so this information was included in a separate section of the summary document. Elements identified to be included in the protocol that presented potential feasibility challenges were also highlighted in the summary document.

The second step involved refining the intervention protocol. The summary document was circulated to the co-production team members, who then worked through the many ideas and suggested changes identified through the stage 2 workshops. Collaborative decisions were made regarding the content of the protocol through facilitated discussions. Summaries of the outcomes of this workshop were provided to attendees with opportunity for amendment. The final outcome from this stage was the intervention protocol prototype, fully described using the TIDieR checklist.

#### Stage 4: adaptation (July 2019)

During this stage adaptations to the intervention protocol were made with the input from a broad range of knowledge user informants. The product of this stage was the intervention protocol and associated participant resources. This stage involved two workshops where different groups of knowledge user informants worked together, i.e., participants in both workshops were people with lived experience of the condition (stroke), caregivers and healthcare workers. One workshop involved mainly knowledge user informants involved in Stage 2 and was held in one Australian state (New South Wales) as this was the lead site for the planned pilot trial of the intervention. The other workshop was run in different state in Australia (Victoria), which was a planned additional site for the pilot trial. Purposive sampling during recruitment was used to ensure a diverse range of knowledge user informants were included.

The first step in stage 4 was to prepare the content for the workshops, including a workshop presentation, draft intervention resources, and demonstration of aspects of the intervention. The second step of stage 4 was preparing the workshops for the knowledge user informants. Similar processes were used from previous workshops to ensure workshops met attendees needs and preferences, logistics such as access, parking and catering were coordinated. Information was sent out to knowledge user informants prior to workshops to provide opportunity for preparation.

The third step in Stage 4 was running the workshops. The workshop formally commenced with the presentation outlining the intervention design project, the aims of the workshop and key elements of the intervention protocol. Aspects of the intervention were then demonstrated. The workshop knowledge user informants then addressed the question “How could the program be improved to suit you?” The workshop facilitator iteratively summarised and confirmed ideas and outcomes to ensure accurate interpretation of knowledge user informant input. The workshops were audio recorded, and summaries were sent to all participants with the opportunity provided to amend the summaries. The final step of stage 4 was to further refine the intervention protocol based on the knowledge user feedback gathered from the workshops.

## Results

Thirty-seven knowledge users (20 healthcare workers, one behaviour change researcher, 11 people living with stroke and 5 caregivers) took part in the co-production process. Five knowledge users formed part of the co-production team, and 32 participated as knowledge user informants. Box 1 highlights how knowledge user input shaped our intervention protocol. The comprehensive intervention protocol developed during the co-production process and, described according to the TIDieR checklist [28] is presented in Additional file 1. Key features include details of resources and equipment co-produced with knowledge users, key information to be provided to intervention participants to facilitate participant safety, the use of telehealth software and equipment, as well as options for tailoring to individual stroke survivor needs and preferences.

### Our exemplar: the development of our intervention of supervised exercise to be delivered via telehealth, aimed at reducing secondary stroke risk

#### Stage 1: start-up and planning

None of our initial co-production team had experience using an IKT approach, so team members reviewed literature on IKT and considered its potential application in the context of the design of a physical activity for people with stroke. Researchers used their networks to invite knowledge users to join the co-production team. One workshop occurred in stage 1 in NSW, in January 2019. This workshop ran for 2 hours (excluding break time) and was attended in-person by co-production team members (MB, HJ, MG, DM, CE and ER) and one researcher on the team attended via videocall (CS). Strategies were put in place to support our stroke survivor member (e.g., provision of information prior to the workshop). We also provided additional supported communication strategies reactively throughout the project when team members identified this need. No formal training was provided to any of the co-production team members regarding collaborative research. However, the IKT approach and expectations were discussed.

During this stage we identified some gaps in skills and subsequently invited a rehabilitation physician (MP) to join the co-production team. We identified the key knowledge user groups that needed to be included the intervention design to be:

- stroke survivors with and without experience in the receipt of telehealth exercise, and with diversity in lived experience.
- caregivers of stroke survivors.
- clinicians with and without experience delivering exercise via telehealth.
- clinicians with experience delivering exercise to stroke survivors.
- healthcare managers.
- healthcare information technology staff working in telehealth.
- rehabilitation physicians and neurologists.

We identified additional knowledge users from the above categories who were recruited (*knowledge-user informants)* and involved through workshops, or a suitable alternative including individual interviews or mini-workshops to optimise participation. Our stroke survivor co-production team member (MB) recommended strategies to optimise engagement with stroke survivors.

The following key *essential components* of the physical activity intervention were confirmed for inclusion in the intervention. These essential components were selected because there was evidence to support their inclusion in the intervention, and were deemed feasible (i.e. there were resources available to ensure the intervention could be piloted):

- Intervention to be at least 6-months duration [29].
  - First 3 months: twice-weekly supervised exercise (via telehealth) incorporating moderate to vigorous physical activity [30–32].
  - Second 3 months: weekly telehealth session to support continuation of physical activity [29, 30].
- All participants would be provided with a Fitbit (Fitbit, Inc., San Francisco, California, USA) to assist them in monitoring their physical activity [33–35].
- The intervention would be delivered via telehealth (video calls) to allow accessibility nationwide [36–38].
- The primary outcome to reflect secondary stroke risk would be blood pressure, as measured by participants twice daily for a week at each assessment point [39, 40].

#### Stage 2: content development

We ran two 90-min face-to-face workshops for stroke survivors and caregivers, this included time for morning or afternoon tea with informal introductions and an opportunity to fill out demographic questionnaires. Numbers at workshops were limited to 9 attendees (6 participants at the first workshop and 4 at the second). Preparations were made to optimise knowledge user informant’s ability and willingness to engage in workshops. This included ensuring appropriate strategies to support people with aphasia (a communication disability) and people with physical disabilities (e.g., providing appropriate set-up of the workshop environment to optimise safety, comfort and interaction);

We also sought input from healthcare workers. We used a flexible approach in response to healthcare workers’ needs to accommodate their work commitments—this meant scheduling individual 30-min interviews (*n* = 4) or shorter (30 min) mini-workshops involving 2 healthcare workers (*n* = 4). Sessions were held in-person, via video-call or phone.

Information, including the types of questions to be addressed in the workshop, were sent to knowledge user informants prior to their workshop. For workshops involving people with lived experience of stroke we included a video created by, and featuring, our co-production team member with a lived experience of stroke. This video explained the project and encouraged knowledge user informants to share their ideas and opinions. The video format was chosen as it enabled our co-production team member with a lived experience of stroke to be involved, while minimising the demands on their time.

During workshops and individual interviews, the facilitator asked questions which encouraged knowledge user informants to identify outcomes which were important to measure, as well as potential barriers and enablers to the proposed intervention. This discussion was used to help participants identify what should be included in the intervention (i.e. the content) to help them decide if they would use the program. Knowledge user informants’ perspectives on the parameters of the intervention being designed, including the aims of the intervention, the intervention itself, and outcomes to measure were gained.

#### Stage 3: intervention protocol development

This stage involved one 105-min workshop. This workshop was attended by two researchers and the four knowledge user co-production team members. Additional key elements such as: options for peer support; optional home visits to ensure a safe exercise environment; a wellness check prior to each intervention session; options for out-of-hours exercise sessions (and recording data on how often this occurred); behavior change training for intervention staff; and an activity diary to be offered as an additional strategy for participants to self-monitor their physical activity were included in the protocol. These additional elements were largely derived from stage two but were clarified, prioritized, adapted or built on by the co-production team who applied their own knowledge and expertise to the intervention development. This helped ensure a cohesive intervention that met the feasibility requirements of our planned pilot trial. For example, stage two workshops identified flexible appointment times were needed to optimize accessibility for stroke survivors. In Stage 3 the co-production team identified sessions outside standard working hours were not feasible in public health services. Therefore, the co-production team decided that appointments would be made available outside business hours (8 am–5 pm) as needed and pending clinician availability, with data on session times recorded to inform future implementation options. The key essential components (identified in Stage 1) remained unchanged, however options to tailor these were added including an additional option for monitoring physical activity beyond the Fitbit (the activity diary), and the option for a home visit (as deemed necessary) to the otherwise entirely telehealth delivered intervention. Areas that required further input from knowledge users were identified so they could be addressed in Stage 4 workshops, including the name of the intervention and the resources to be used as part of the intervention. Three additional people also contributed to the intervention design at this time via an individual interview and a two-person mini-workshop (two existing co-production team members unable to attend the first workshop and a knowledge user informant recruited as part of Stage 2).

#### Stage 4: adaptation

The first workshop ran in Victoria, Australia, and was attended by eight knowledge user informants (3 stroke survivors, 2 caregivers and 3 healthcare workers). The second was run in New South Wales, Australia, and was attended by six knowledge user informants (3 stroke survivors, 1 caregiver, and 2 healthcare workers). Three or four members of the co-production team were also present at each workshop in a research capacity. One clinician knowledge user participant attended via videocall for each workshop, everyone else attended in person.

The workshops ran for approximately 90 min, with additional time for informal introductions in the setting of a morning or afternoon tea and time for filling out demographic questionnaires. Within the workshops the telehealth videocall process was demonstrated, and equipment and draft resources to be used were available for knowledge user informants to review. Examples of knowledge user informants’ ideas or opinions that were used to adapt the program included: changing to the formatting of the intervention’s resources to improve their accessibility for people with aphasia (a communication disability); binding the written resource to make it more accessible for people with impaired arm or hand movement; and further options for participant support (such as audio or video alternatives to written communication, and opportunities to decline or accept session reminders). The re-naming of the program also occurred in Stage 4 with input from our knowledge user informants.

Box 1: How knowledge user involvement shaped our intervention

**Table:** 

Key co-production team knowledge user contributions arising from Stage 1	
Stroke survivor knowledge user on the co-production team	Clinician knowledge users on the co-production team
Brought lived experience of the challenges of participating in research to inform the research approach Recommendations for workshop structure (e.g., time limits, pacing, language, input into the presentation of the program in Stage 4) Developed an introductory video for stroke survivor/carer workshops (Stage 2) describing the project and highlighting the role of stroke survivors	Brought knowledge and experience regarding the context that healthcare workers participating in the project are working within to inform the research approach Identified potential key healthcare worker knowledge users to be involved in the co-production Prioritised early involvement of knowledge users to optimise their contributions and ‘buy in’

Key protocol elements arising from knowledge users’ input (Stages 1–4﻿)^a^	
Suitable patient resources (information booklet and prompt sheet) including prioritized content, aphasia friendly format, easy handling design and storage Increased options to tailor support to individual stroke survivor needs including those with visual or communication impairment, and with varying confidence using technology (e.g., options for online video/images instructions or paper-based images/instructions) Inclusion of a peer support option Tailoring of physical activity monitoring—e.g., options to use an activity diary or their own activity monitor (if preferred) in addition to a Fitbit™ Re-naming the program to reflect what it means to stroke survivors (i-REBOUND, Let’s get moving!) Option to provide home visits to provide extra support to participants who require it (e.g., for technology or home set-up) Increased emphasis on education regarding information about when not to exercise and advice regarding measures to increase safety if exercising alone Extra initial session added at the beginning of the intervention to facilitate effective assessment, build rapport, and troubleshoot Monitoring participant’s preferences for session times to determine if they are in line with the public healthcare workforce needs Highlighted pre-requisite training/knowledge/experience important for clinicians undertaking the role Support from local interpreting service Identified potential equipment needs for the program Increased time allowance pre- and post-exercise sessions for admin/troubleshooting	

^a^Through collaborative decision-making knowledge users on the co-production team participated in prioritising these within our intervention protocol.

### Challenges of the process

To complete our practical guide for researchers co-producing interventions for clinical trial we will now present the challenges of the 4-stage process.

This first workshop highlighted that time and preparation are required to help mitigate the significant challenges involved in creating genuine partnership within the co-production team. Throughout all stages of the project, there was an ongoing tension to balance the competing time pressures of clinicians, researchers, and people with lived experience of stroke. The co-production team’s preference for in-person workshops to optimise communication required interstate travel for the workshop facilitator. This limited the feasibility of frequent in-person workshops. Less frequent, longer duration meetings with quick decision-making processes was the preferred way of working for clinician and research co-production team members. However, shorter but more frequent workshops and more time allowed for decision making better met the needs of our stroke survivor co-production team member. The time from the first workshop to the completion of the project was approximately seven months. Initially, we had estimated the process may take approximately four to five months, however, we ensured a flexible timeline to allow for potential delays related to recruitment, ethics approval. Potentially, the project could have been completed in less time, however this would have meant fewer and a less diverse group of knowledge user informants which we feel was important to the success of our project.

An implicit power imbalance exists between people with the lived experience of receiving healthcare or research interventions (e.g., a patient) versus those delivering it (e.g., healthcare provider or researcher). Additionally, people with lived experience of a health condition are less likely to speak medicalized or research language, which could inhibit their ability to participate in collaborative decisions if a common language is not applied throughout the project. By only involving one stroke survivor on our co-production team, we inadvertently risked burdening her more than the other co-production team members because our stroke survivor member was the only person able to provide input from her perspective as both a stroke survivor and someone without formal research training or a health professional qualification.

Time management and clear communication to all members of the team was challenging. It takes time and skill for clinicians and researchers to explain complex concepts in plain, jargon-free language to other knowledge user informant groups. Researcher and clinician co-production team members needed to adapt their communication style to ensure discussions were accessible to other team members. Beyond using appropriate language, team members also needed to ensure they provided adequate time for cognitive and language processing, which was particularly important for our stroke survivor team member. Furthermore, at the first workshop, considerable time was spent educating all members of the co-production team about the key concepts of the IKT approach adding to time pressures.

Optimising knowledge user informants’ input was a challenge throughout all the workshops. Time limits on workshops are necessary to ensure knowledge users can contribute meaningfully, without undue burden. However, shorter workshops make it difficult to cover the essential information and allow for in-depth discussions. Stage 3 (intervention protocol development) was the most challenging to manage issues around competing time pressures and conflicting needs of knowledge users and researchers on the co-production team. The workshop duration was too long for our stroke survivor team member and the preparation material too extensive, which initially limited her ability to provide input into all aspects of the design. We adapted to this team member’s needs by developing more accessible preparation material and having an additional meeting with this team member. Managing time pressures and the opposing needs of different team members made genuine collaborative decision-making challenging. We must acknowledge that the ideal process does not exist, and that honest and open communication across all co-production team members was essential to identify areas that need improvement so they can be addressed.

### Practical tips for researchers applying our approach to co-produce an intervention ready for clinical trial

#### Stage 1: start-up and planning

- *Establish the team*, when doing so engage more than one person per key knowledge user informant group on the co-production team to allow sharing of the role to provide a diversity of opinion. This is of particular importance for the co-production team members with lived experience of the health condition to address potential power imbalances. Having two or more people representing the key knowledge user informant groups allows for sharing and flexibility of roles which may be particularly important for knowledge users with complex health conditions or limited flexibility in their professional or personal commitments. We also recommend the team ensure they have adequate resources (e.g. time and funds for remuneration) to support additional team members.
- *Building a cohesive* team by avoiding the use of jargon and investing time prior to the first meeting to develop relationships between all co-production team members, in particular those without experience in research.
- *Establishing processes and ways of working*. This requires clarifying co-production team member roles as early as possible (with the understanding that the iterative nature of co-production there is scope for these to change throughout the process),
- *Ensure clear and consistent expectations* are established within the co-production team regarding the research process. Consider providing education (formally or informally) to all co-production team members about the IKT approach, the intervention being designed, and what to expect prior to the initial workshop if they are new to research or the IKT approach. Document the planned processes with a terms of reference document.
- *Ensure a shared understanding of the principles of research partnership*. Hoekstra et al. identifies overarching principles in their systematic review [41]. However, there is no universal checklist of principles. Instead, it is the meaningful engagement of knowledge users in collaborative decision making in an environment of mutual respect, fundamental to IKT, that lay the foundations for the team to determine their specific principles.
- *Consider the potential benefits of engaging an independent workshop facilitator* who is not invested in the project or an expert in the field to assist the co-production team to share power and achieve a common language.
- *Ensure access issues are discussed and supported* with appropriate strategies prior to the first workshop. Such issues include communication and transport needs, and remuneration of costs.

#### Stage 2: content development

- *Provide information regarding the aims and content of the workshop* to knowledge user informants prior to workshops so they can prepare in advance.
- *Consider running a series of workshops* where the first group meeting is focused on information provision only (as resources allow).
- *Provide flexibility* in the delivery and timing of workshops.

#### Stage 3: intervention protocol development

Tips provided in Stage 1 are relevant to supporting the success of the Stage 3 workshop, additionally:

- *Ensure the workshop format and timing will address the co-production team’s diverse needs*. This is likely to vary between individual knowledge users and we therefore recommend communicating with knowledge users to determine this. We found our in-person workshop was too long for our team and limiting it to 90 min would have been preferable. Teams should consider whether this workshop could be split into two parts and whether some decisions could be made asynchronously (e.g. via email discussion) or remotely (e.g. via videoconferencing).
- *Consider the need for additional preparation* for people with lived experience of a health condition on the co-production team to ensure they are adequately informed, and their access and communication needs are met prior to the workshop.
- *Ensure all co-production team members have the opportunity and confidence to provide feedback* on the project’s research processes and how they can be improved and ensure there is flexibility within the project design to act on this feedback.

#### Stage 4: adaptation

Practical tips outlined in *Stage 2* are relevant again in this stage. Additionally.

- *Include a mix of knowledge user groups in workshops* to allow different knowledge user informant perspectives to be presented and collaborative adaptations made**.**
- *Include participants from Stage 2* in as they bring prior knowledge of the project.
- *Allow sufficient time for questions and clarification* throughout the workshop so knowledge user perspectives can be clarified in a common language to all knowledge user informants.
- *Provide preparation materials tailored to knowledge user informants’ needs and preferences* prior to workshops to help bridge the knowledge gaps between different knowledge user informant groups.

Box 2 provides a summary of the key challenges and recommendations for applying an IKT approach to intervention design. We recommend these in the context of the design of complex interventions for clinical trial. However, collaborative research approaches more broadly have reported similar challenges including time; a lack of dedicated resources; geographical distance; a lack of knowledge or skills in IKT; diverse needs and priorities of knowledge users; sharing of power; and overburdening of knowledge user informants in the co-production team [41, 42] Therefore, these challenges and tips may be relevant to IKT approaches more broadly.

Box 2: Challenges and top tips for researchers when using an IKT approach

**Table:** 

Key challenges of IKT intervention design	
Meeting the needs of a diverse range of knowledge users and employing a ‘common’ language Researchers facilitating and participating concurrently in workshops Ensuring ethical approvals and amendments are gained in a timely manner (given the iterative nature of IKT) Ensuring co-production team members are adequately prepared/trained Accommodating competing demands, including: – Time pressure to complete project – Real or perceived power imbalance – Minimising time burden of knowledge user	

Top Tips for:	
**IKT intervention Design**	
Initiate the working relationship of the IKT partnership as early as possible Identify early any training needs to ensure adequate support of knowledge users Ensure the co-production team has an understanding and expectation of ‘shared power’ for the project Ensure adequate inclusion of knowledge user groups to facilitate ‘equal voice’—i.e., at least two knowledge users included for each knowledge user groups in your co-production team Encourage reflection and feedback and formally evaluate it Provide flexible approachs to workshops to optimise participation: e.g., alternatives to larger workshops such as individual interviews, mini-workshops Ensure knowledge users are comfortable to express their views within workshops: for example, light refreshments 15 min prior to facilitate an informal introduction, knowledge user delivered summary of the project Build mutual respect and shared power to facilitate respectful discussion/resolution of differences of opinion/knowledge disparities	
**Researchers collaborating with knowledge users﻿**	
Find the right people for your project—consider prior, knowledge, skills, and experience Invest time to create a genuine connection and build working relationships through social and informal interactions e.g., catch up for coffee Ask about what support people may need to ensure they are able to contribute. What accessibility needs do they have? Work out the role of the knowledge user in your project and write it down. Have open discussions about roles and how they may change over time Allow adequate time for preparation (including time to read documents prior to workshops/meetings) Provide opportunities for feedback after workshops/meetings and be open and responsive to any feedback provided Develop communication strategies that are inclusive and work for everyone on the team: e.g., lay person summaries at the top of emails can be useful Ensure adequate funding to support consumers (e.g., as a paid member of the co-production team, or to reimburse time spent on the project)	
**Knowledge users collaborating with researchers﻿**	
Remember the research process can be tricky to understand, but this is not a requirement. You are an equal member of the team with specific expertise Communication is key. You may choose one person on the team as your go-to for feedback and/or support during the research process At the beginning of the project tell your team what you need. This may include anything you need to be able to contribute fully to the process (e.g. duration or timing of meetings, transport, breaks, methods of communicating). If you don’t know or have never been in this situation then that is ok, you can make changes as you go Make sure you understand your role/s in the project and any payments or compensation you will receive, and write it down

## Reflections

We have provided a practical guide for researchers on how to co-produce an intervention ready for clinical trial. We provided an exemplar based on our experience, highlighted the challenges we faced and provided practical tips for others embarking on this process. Our co-production team deem the early involvement of knowledge users within the co-production team as critical in guiding the development of a more broadly acceptable intervention (including for stroke survivors with visual impairment, aphasia, and those from culturally and linguistically diverse backgrounds). We feel the key elements, adaptations, or refinements resulting from knowledge user input (Box 1) will play a crucial role in the success of the intervention. We feel the process was a worthy investment of resources. However, throughout this process we identified some broader challenges of intervention co-production that warrant further discussion.

### Power and leadership

By working in partnership with knowledge users with lived experience (such as healthcare workers and people with health conditions), knowledge users can apply their expertise [9] to ensure outputs meet the intended users' needs, an essential element for successful implementation. Developing and maintaining the researcher-knowledge user partnership where power is shared within the co-production team requires a significant commitment by its members and can present challenges [13, 43]. The sharing of power can be a daunting prospect for researchers, because most researchers have been educated in a culture where researchers determine all aspects of the research process, from defining the research question through to dissemination. Jargon-free communication is important to avoid reinforcing power differentials and to optimise understanding [44]. Sharing power is made even more challenging in the absence of systems to support authentic collaboration [45] and the need to deliver the research in a timely manner.

Collaborative decision-making is an essential element of partnership. It is important to highlight that collaborative decision-making does not require complete consensus from co-production team members on all decisions. However, it does require leadership to ensure all co-production team members have the opportunity to be heard and respected in decision-making processes. Consistent with previous projects applying an IKT approach, we observed disproportionate participation or engagement by knowledge user members of the co-production team at different stages [11, 46]. Sibbald et al. highlight that varying levels of contributions by knowledge users and researchers at different times throughout a project may actually indicate “a more nuanced characteristic of these partnerships” [47]. However, for this to be true, it is critical that a lack of involvement in certain phases of the project are not the consequence of a lack of opportunity. We aimed to optimise the use of the co-production team’s time by identifying a priori key decisions to be addressed at workshops. However, the determination of what constitutes a key decision is subjective. Researchers in the co-production team were spending the majority of the time on the project, therefore the identification of the key decisions and leadership within the co-production team fell to them.

### The co-production team and broader knowledge user engagement

Our project involved 37 knowledge users (five co-production team members and 32 knowledge user informants), and accordingly different knowledge users had different levels of engagement with the project. We employed the principles of mutual respect and partnership by setting expectations around respectful conversation, valuing everyone’s ideas, and including the ideas raised in workshops throughout all stages of the project. However, the co-production team (comprising both knowledge user informants as well as researchers) defined the research approach and developed the intervention protocol prototype and therefore undertook most of the decision-making, following consideration of suggestions and feedback from knowledge-user informants.

Our experience identified that the preparation needed for effective engagement with knowledge users may need to be tailored specifically to their needs and should be identified early. Evidence now suggests that health care professionals require ongoing training (such as communication partner training) to support communication for people with aphasia [48], yet none of our co-production team participated in this training. Had specific communication support strategies been identified and implemented at the initiation of the project (e.g., through individual, in-person meetings with co-production team members to specifically identify strategies, and training of other research team member in communication partner training) some of the challenges around communication may have been avoided.

### Building working relationships to support partnership

Strong relationships are fundamentally important for working collaboratively with stakeholders [13, 45, 49]. IKT research highlights the importance of an initiation phase when applying an IKT approach to establish collaborative working partnerships, which can take between six months and six years to develop [43]. Just as good friendships take time to become established and mature, so too do productive working relationships, especially when working with people from different backgrounds and with different perspectives and skillsets. Our experience also highlighted that an informal environment can facilitate a working relationship with knowledge users without previous research experience. Our tight 7-month timeline required a condensed initiation period. This was facilitated by a small co-production team, a clearly defined project, and a partnership that utilized pre-existing relationships.

### Ethics

Ethics approval of research is necessary to ensure research is safe, appropriate, and carried out to a high standard. However, when working collaboratively with different knowledge user informant groups, ethical requirements themselves can present challenges [50]. For instance, flexibility and responsive iterations which are often preferred in collaborative projects can be inhibited by pre-specified procedures required for ethical approval [50]. In Australia, named investigators are routinely required to complete ethical research practices (e.g., Good Clinical Practice certification), regardless of their prior research training or educational qualifications. When training is not suited to knowledge users’ needs or educational backgrounds and is not relevant to knowledge users’ roles in the research, it can place undue barriers on knowledge user’s engagement in research teams. Therefore, the requirement that named investigators complete generic training packages (developed for research academics) may need to be challenged to ensure acceptable and accessible pathways for knowledge users in research.

### Strengths and limitations of the project

Our project was occurred in two Australian states and involved knowledge users in stroke, telehealth, behaviour change and health management. We anticipate the process we have described will be transferable to other teams seeking to co-produce interventions for clinical trials in other clinical areas and other geographical regions, however further research is required validate this. The co-production formed part of a larger trial [24] addressing a known research gap. However, this approach could potentially be applied to identifying research gaps with the engagement of key knowledge users such as policy makers, consumers and healthcare workers.

A lack of evaluation of our co-production process limits the ability to draw conclusions on the long-term impact of the IKT process. We recommend future research embed evaluation of the co-production process to evaluate the partnership and the impact of the co-production on the success of the intervention. Our co-produced intervention is currently being tested for safety and feasibility through a pilot randomized trial [24].

## Conclusion

Co-production of complex interventions for clinical trial should ensure they meet the needs and preferences of knowledge users. The IKT approach described provides practical guidance and structure to support broad knowledge user informant engagement in an iterative intervention co-production process that prioritises partnership and collaborative decision making. Importantly, we have described the challenges experienced when working this way and identified strategies that may be used to help mitigate these. We anticipate that the process described in this paper will be of assistance for others seeking to co-produce complex interventions for clinical trial.

## Supplementary Information

**Additional file 1: Table S1.** Intervention protocol mapped to the TIDieR Checklist.

## Data Availability

Data generated during this project are included in this published article [and its Additional files].

## Glossary

IKT: Integrated knowledge translation
Co-production team: authors MB, CE, EL, ER, CS, AP, MP, DM, HJ, MG, IG.
Knowledge user informants: people recruited to participate in workshops and individual interviews
Knowledge user: a stakeholder that will use and apply the research in the future
