# Supplementary material for: Fit for purpose. Co-production of complex behavioural interventions. A practical guide and exemplar of co-producing a telehealth-delivered exercise intervention for people with stroke
Source: Health Res Policy Syst. 2022 Jan 3;20:2. doi: 10.1186/s12961-021-00790-2 (PMC8722305; doi:10.1186/s12961-021-00790-2)
Supplement: Supplementary file 1 — Additional file 1: Table S1. Intervention protocol mapped to the TIDieR Checklist. [file 12961_2021_790_MOESM1_ESM.docx]

Table 1: Intervention Protocol populated into the TIDieR Checklist^1^

| TIDieR item | Description |
| --- | --- |
| **1. Brief Name**  Provide the name or a phrase that describes the intervention | i-Rebound after Stroke. Let’s get moving! |
| **2. Why**  Describe the rationale, theory, or goal of the elements essential to the intervention | **Rational:**  Secondary stroke rates are high, while most strokes are preventable.  Adequate physical activity can reduce stroke risk, yet many stroke survivors have low levels of physical activity. Furthermore, physical activity can help stroke survivors feel and move better.  Advice and education to exercise or increase physical activity in isolation may not be effective to increase physical activity after stroke. However, lifestyle interventions which involve exercise have been found to reduce stroke risk factors.  There are no large-scale trials that have looked at the effectiveness a program of supervised exercise to increase physical activity after stroke.  **Goal:**  To support stroke survivors across Australia to reduce their stroke risk, through increased physical activity in line with recommendations of a minimum of150 minutes per week of moderate to vigorous physical activity.  **Essential elements:**  Supervised exercise sessions (3 months).  Support to maintain increase physical activity levels (3 months).  Intervention to be delivered via telehealth.  Embedded behaviour change strategies to support long-term uptake of the intervention. |
| **3. What: Materials**  Materials: Describe any physical or informational materials used in the intervention, including those provided to participants or used in intervention delivery or in training of intervention providers. Provide information on where the materials can be accessed. | Starter Pack, including   - Mesh bag to keep equipment in (able to be opened using one hand) - Information booklet, A4 size, colour, spiral bound, hard copy (and option for PDF), designed to meet the needs of people with communication deficits (aphasia), key information including:   - Aims of the program and what it involves   - How to prepare for telehealth sessions   - Phone number for assistance   - Brief summary of types of exercises involved in the program with images of a stroke survivor demonstrating   - Equipment used for exercise and monitoring   - Information on when not to exercise - Monitoring equipment   - Pulse oximeter   - Laminated page with Borg rating CR-10 scale optimised for participants with aphasia and instruction to prepare for exercise session on the other side   - A Fitbit^TM^ wrist-worn activity tracker   - BP monitor   Telehealth equipment:   - Technology to host telehealth sessions:   - Appropriate device e.g. laptop or tablet (smart phone alone not adequate due to small screen size) - Internet connection, adequate to support video calls - Teleconferencing software - Optional additional equipment to be considered to meet participant needs, e.g. stylus, tablet holder, portable webcams (+/- blue tooth), option to mirror session to TV screens, speakers   Exercise equipment:   - Prioritise equipment that is readily available in participant homes- e.g. tins of food (to be used as weights), stable chair, bench - Consider option to hire or provide equipment if needed (e.g. unable to achieve required intensity of exercise without additional equipment) - Exercise equipment that optimises opportunity to continue with the program after cessation of the intervention   Optional materials, to be provided (as appropriate) according to participant needs or preferences:   - Activity diary - Home exercise programs (e.g. written or emailed), individualised to participants- these may also include information regarding exercise intensity when not to exercise - Schedule of appointment times (e.g. a timetable or email confirming times for regular weekly appointments) - Videos of exercises accessed online* - Other online resources to support participation (e.g. instruction videos to support use of equipment)   Equipment to support outcome measures of secondary stroke risk including:   - Blood pressure monitor- primary outcome measure - ActivPal – physical activity monitor - Surveys/Questionnaires/Assessments |
| **4. What: Procedures**  Procedures: Describe each of the procedures, activities, and/or processes used in the intervention, including any enabling or support activities | **Clinicians** are familiar with the policy which guides management of medical emergencies  **Participants** of culturally and linguistically diverse backgrounds will be offered interpreting services  **Prior to participation**:   - Participants will require medical clearance (e.g. from their GP) prior to participating   - Clearance form will highlight potential contraindications to the program - Participants will be sent the *Starter Pack*   **Initial telehealth exercise session** (approx. 1hr duration) aims to include:   - Clinician subjective and/or objective assessment** to gain relevant information regarding:   - Exercise environment considering appropriate space for exercise, lighting, privacy, noise, temperature, falls risk (e.g. trip hazards)   - Exercise equipment available at home   - Position to place the device (e.g. laptop or tablet) for exercise sessions   - Medical history   - Level of function (e.g. gait, sit to stand, falls risk), and any relevant impairments (e.g. strength, sensation, balance)   - Other relevant information such as any cognitive, or communication deficits   - Assistance/support needed and availability for sessions   - Preferences for communication and/or modes of contact (e.g. email or post)   - Preferences for session reminders (e.g. SMS or email)   - Wellness check: informed by the medical history information, to also include (as able) heart rate, oxygen saturation, and participant’s self-reported wellness.   - Need for home visit: conducted at the discretion of the clinician if deemed necessary to ensuring participation in the trial - Education/advice regarding   - The i-REBOUND Let’s get moving program   - The telehealth videoconferencing system -troubleshooting as required, including:     - How to disconnect from sessions, mute/unmute, and turn camera on/off   - The Borg CR-10^2^ rating of perceived exertion scale   - The pulse oximeter   - Options for self-monitoring physical activity including the Fitbit^TM^ and/or activity diary   - Exercise safety, including when not to exercise   - Option for participation in peer support   - Physical activity benefits - Discussion (as time allows) to be continued throughout the supervised exercise and support session regarding participant’s:   - Current physical activity level, preferences, potential barriers and facilitators, and perceived pros and cons   - Physical activity targets (initial goal setting) and strategies to increase physical activity - Optional (as time allows and if appropriate) an introduction to one of the exercises to form part of the participant’s program   **Supervised exercise sessions** (months 1-3), occur twice weekly, of approximately 45min duration, will include:   - Wellness check (including as able heart rate, oxygen saturation, and questions such as are you feeling well, any falls/injuries/illness/pain or discomfort since the previous session?) - Supervised exercise (targeting cardiorespiratory fitness including some exercises involving resistance (e.g. light hand weights) including:   - Warm-up (approximately 5-minute duration)   - 20 minutes of moderate to vigorous physical activity     - Use a standard routine of exercises- progressed as appropriate by the treating clinician to increase exercise dosage     - Exercises may include marching or stepping on the spot, moving from sitting to stand (sit to stand repetitions), lifting small weights or walking, and will be adapted to suit participant’s ability and fitness level   - Cool down (approximately 5-minute duration)   **Exercise Support Sessions** (approximately 30min duration) months 4 to 6 including:   - Wellness check (including questions such as are you feeling well, any falls/injuries/illness/pain or discomfort since the previous session?) - Once weekly clinician support for an individually tailored home and/or community physical activity program which may include:   - Continuing the in-home exercise sessions, attending local stroke-specific or general exercises classes in the community, or walking groups   Both **supervised exercise** and **exercise support** sessions will include:   - Talking about ways to keep active incorporating behaviour change techniques^3^ , including:   - Reviewing self-reported physical activity as measured by the participant (e.g. via Fitbit^TM^ activity diary)   - Setting / reviewing activity targets (goals)   - Advice regarding suitable exercise/activities outside of supervised exercise sessions (where appropriate)   - Individual barriers and facilitators to physical activity and strategies to overcome these - Additional time in sessions to for technical troubleshooting,   **Clinicians:**   - Structure sessions to minimise any need to adjust technical equipment - Involve family or support people in the set-up of the telehealth/equipment as needed - Join in the supervised exercise as able - Provide feedback to participants - Allow sufficient time between session for appointment booking and documentation - Prioritise consistency of therapist (as able) - To record:   - Technical issues, how they were resolved, and time spent addressing these   - Reasons why session did not run to plan   - Duration of exercise sessions   - Time spent in moderate to vigorous physical activity each session   - Participants’ activity targets   - Participant self-reported physical activity outside session times   **Participant** will be asked to track their activity each week using and activity tracker (e.g. Fitbit^TM^) and/or and activity diary  **Peer support** to be offered to participants and implemented as appropriate, may include:   - Closed Facebook group |
| **5. Who Provided**  For each category of intervention provider (such as psychologist, nursing assistant), describe their expertise, background, and any specific training given. | **Physical activity intervention** provided by clinician (physiotherapist, exercise physiologist or exercise scientist) with:   - Knowledge of stroke and experience delivering exercise to stroke survivors - Trained in healthy conversation skills^4^ - Skills or knowledge required to optimise the intervention delivery:   - Strong communication skills   - Knowledge of telehealth etiquette - Experience in delivering exercise via telehealth, or, support and/or training provided by a clinician experienced in supervised exercise delivered via telehealth.   **Assessment:**  Conducted by blinded assessor |
| **6. How**  Describe the modes of delivery (such as face to face or by some other mechanism, such as internet or telephone) of the intervention and whether it was provided individually or in a group | Generally, all telehealth exercise session will be delivered individually  Both supervised exercise and exercise support session will be delivered via **telehealth**  using video calls (teleconferencing software) where able. Telephone may be used if appropriate when videocalls are not available and telephone delivery is deemed appropriate by the clinician.  **Other support** as negotiated with the participant according to their preferences may include text messages and email so support increased physical activity (e.g. appointment reminders, home exercise programs).  **Optional peer support:** Via a closed Facebook group |
| **7. Where**  Describe the type(s) of location(s) where the intervention occurred, including any necessary infrastructure or relevant features | The intervention will be delivered via telehealth. With clinicians in their place of work and participants in their own homes or a suitable alternative (e.g. relative’s house or workplace if deemed appropriate).  **Participant requirements:**  Internet connection, a device (desktop, laptop, computer tablet) with webcam and microphone capability – tablets available for loan if the participant does not have an appropriate device, suitable environment to exercise (assessed in initial telehealth session).  **Clinician requirements**   - Internet connection and device with webcam and microphone capability and suitable place to deliver exercise sessions from (considering space, privacy, lighting, noise) |
| **8. When and How Much**  Describe the number of times the intervention was delivered and over what period of time including the number of sessions, their schedule, and their duration, intensity, or dose | **Initial telehealth session (x1)** occurs after randomisation and prior to the first telehealth supervised exercise session. Session duration approximately 1hr.  **Supervised exercise sessions, dosage, and intensity:** 3 months (months 1-3), occur twice weekly for a total of 24 sessions:   - Warm-up approximately 5 min - 20 min of moderate to vigorous physical activity   - Generally, no vigorous intensity exercise in the first 2 weeks   - Some participants may need to build up to the 20 min of exercise or require rests between exercises - Cool-down approximately 5 min - Overall session duration approximately 45min   **Exercise Support Sessions, dosage: 3** (months 4 to 6), 12 sessions occurring weekly, session duration approximately 30min. |
| **9. Tailoring**  If the intervention was planned to be personalised, titrated or adapted, then describe what, why, when, and how | To optimise the acceptability and safety of the intervention the following aspects will be individually tailored to participants’ needs, ability, and preferences by clinicians throughout the intervention period:   - Exercise prescribed - Caregiver involvement - Prompts, reminders, support strategies and materials/resources (including provision of support materials via audio of video) - Activity targets - Session times, appointments will be available outside business hours (8am-5pm) as needed (pending clinician availability) with data on session times recorded |
| **10. Modifications**  If the intervention was modified during the course of the study, describe the changes (what, why, when, and how) | Not applicable. |
| **11. How well: Planned**  Planned: If intervention adherence or fidelity was assessed, describe how and by whom, and if any strategies were used to maintain or improve fidelity, describe them | Fidelity data will be collected at each intervention session including;   1. Session delivery: attendance, duration, adherence to structure, technology fidelity, a therapist survey of perception of participant engagement with each session (five-point Likert scale), and behaviour change techniques applied. 2. Exercise dosage, including: duration, heart rate and rating of perceived exertion during supervised exercise; and self-reported physical activity   In addition, a selection of intervention sessions will be recorded and audited by coding according to Michie’s taxonomy of behaviour change techniques.^3^ |
| **12. How well: Actual**  Actual: If intervention adherence or fidelity was assessed, describe the extent to which the intervention was delivered as planned | N/A  Intervention is currently in pilot stage. |

* Initially to involve trial staff

** some aspects of assessment will rely on self-report due to the remote nature of the assessment

1. Hoffmann TC, Glasziou PP, Boutron I, et al. Better reporting of interventions: template for intervention description and replication (TIDieR) checklist and guide. *Bmj.* 2014;348:g1687.

2. Borg G. *Borg's Perceived Exertion and Pain Scales.* United States of America: Human Kinetics; 1998.

3. Michie S, Atkins L, West r. *The Behaviour Change Wheel. A Guide to Designing Interventions.* Great Britain: Silverback Publishing; 2014.

4. Lawrence W, Black C, Tinati T, et al. 'Making every contact count': Evaluation of the impact of an intervention to train health and social care practitioners in skills to support health behaviour change. *J Health Psychol.* 2016;21(2):138-151.
